# Association of *DRD2* and *BDNF* Genetic Polymorphisms with Exercise Addiction

**DOI:** 10.3390/ijerph22091356

**Published:** 2025-08-29

**Authors:** Izadora Moreira da Silva, Caleb Guedes Miranda Santos, Camilla Geyer de Rezende, Victor Corrêa Neto, Alexandre Palma

**Affiliations:** 1 Escola de Educação Física e Desporto, Universidade Federal do Rio de Janeiro, Campus do Fundão, Rio de Janeiro 21941-599, Brazil; victor.neto@estacio.br (V.C.N.); alexandre.palma@eefd.ufrj.br (A.P.); 2 Teaching and Research Division, Instituto de Biologia do Exército, Rio de Janeiro 20911-270, Brazil; guedes.caleb@eb.mil.br (C.G.M.S.); camillagrezende@gmail.com (C.G.d.R.)

**Keywords:** genetics, exercise, genetic polymorphism, addictive behavior

## Abstract

Exercise addiction is described in the literature as a compulsive behavior associated with adverse health symptoms. Currently, knowledge about the biological and social factors that trigger the development of this behavior is still lacking, and there are no published studies on genetic variants associated with the disorder. Because of this, we genotyped specific polymorphisms in the genes *DRD1* (rs265981), *DRD2* (rs1800497), *BDNF* (rs6265), *HFE* (rs1799945), *ACTN3* (rs1815739), *PPARA* (rs4253778), *PPARGC1A* (rs8192678), and *AMPD1* (rs17602729) to investigate whether they were associated with exercise addiction. In total, 469 men and women, comprising athletes and non-athletes between the ages of 18 and 50, were enrolled in the study. Each participant provided an oral swab sample for genetic analysis and completed the Negative Addiction Scale questionnaire that tests for physical exercise addiction. For the *DRD2* polymorphism, there was a significant association of the *GG* genotype with asymptomatic participants and of the *AA* genotype with participants symptomatic for exercise addiction. Additionally, for the *BDNF* polymorphism, the *CC* genotype was associated with symptomatic participants, and the *T* allele was associated with asymptomatic individuals. However, all associations were found by evaluating the SNP individually, and this demonstrates the difficulty in studying variables related to behavioral phenotypes.

## 1. Introduction

Physical inactivity is considered a risk factor for different metabolic, cardiac, and mental health disorders [1,2]. However, despite the potential benefits associated with the practice of exercise, excessive physical activity can trigger exercise addiction, leading to negative withdrawal symptoms such as depression, anxiety, and irritability [3,4,5]. Although the association of genetic polymorphisms with physical/sport performance and levels of exercise practice has been reported in the literature [6,7,8,9,10], to the best of our knowledge, there are no published studies in which genetic polymorphisms and exercise addiction have been correlated.

Exercise addiction is described as an obsessive–compulsive behavioral change that is associated with symptoms of compulsion, distress, mood swings, anxiety, and depression, with social and personal implications [3,4]. Currently, not much is known about the causes of exercise addiction, with some researchers suggesting increased dopamine levels during and after exercise as a possible etiologic factor, considering the participation of this neurotransmitter in the reward and mood systems [5,6,7,8,9,10,11].

However, other types of compulsive behaviors, such as addiction to nicotine, alcohol, and other psychoactive drugs, were previously associated with different gene polymorphisms [12,13]. For example, polymorphisms of genes that encode dopamine receptors, such as those of the *DRD2* gene, have been associated with the development of addiction to different drugs and psychoactive compounds [14,15]. It has been suggested that the *A* allele, which can decrease the expression of dopamine receptors and increase dopamine levels, is associated with an increased risk of developing these compulsive behaviors [14,15].

Several genes have already been identified as being associated with physical exercise performance and levels, namely, dopamine receptor D1 (*DRD1*) and D2 (*DRD2)*, brain-derived neurotrophic factor (*BDNF*), peroxisome proliferator-activated receptor alpha (*PPARA*), adenosine monophosphate deaminase 1 (*AMPD1*), homeostatic iron regulator (*HFE*), peroxisome proliferator-activated receptor gamma coactivator-1 alpha (*PPARGC1A*), and actinin alpha 3 (*ACTN3*). Given the scarcity of studies on the association of genetic variants with exercise addiction, the objectives of this study were to map the main polymorphisms in the eight genes mentioned above and to investigate whether they were associated with exercise addiction.

## 2. Materials and Methods

### 2.1. Samples

In total, 469 men and women (athletes, exercisers, and sedentary individuals) between 18 and 50 years of age participated in the study and were anonymously coded. All individuals signed an informed consent form for study participation. The exclusion criteria included being under 18 years of age, being pregnant, having any self-reported chronic diseases, or being over 65 years of age. This study was approved by the Research Ethics Committee of the Clementino Fraga Filho University Hospital of the Federal University of Rio de Janeiro (CAAE: 37346714.4.0000.5257).

### 2.2. Data Collection Procedure

#### 2.2.1. Exercise Addiction

The Negative Addiction Scale questionnaire, translated into Brazilian Portuguese by Rosa et al. [16], was completed by each participant to quantify their degree of exercise addiction. Each item was given a score of 0 or 1, totaling a maximum of 14 points. Addiction symptoms were classified into two levels: (i) asymptomatic (0 to 4 points) or (ii) symptomatic (≥5 points). A 5-point cutoff was used, according to Hailey and Bailey [3].

#### 2.2.2. Genotyping

Genomic DNA was extracted from oral epithelial cell samples by carrying out alcohol precipitation, column purification, and membrane filtering steps with the commercial QIAamp DNA Investigator Kit and QIACube automated extraction platform (Qiagen, Valencia, CA, USA). The quality and quantity of the nucleic acid obtained were checked using a NanoDrop 100C spectrophotometer (Thermo Fisher Scientific, Wilmington, DE, USA). All DNA samples were stored at −20 °C before analysis.

Single-nucleotide polymorphism (SNP) genotyping was performed by allelic discrimination assay using TaqMan probes and real-time polymerase chain reaction (qPCR), and the data were acquired on the StepOnePlus™ Real-Time PCR System (Thermo Fisher Scientific, Foster City, CA, USA). The commercial oligonucleotides and probes used for the assays were functionally tested and validated (Thermo Fisher Scientific, Foster City, CA, USA) from the codes of each polymorphism available on the dbSNP and 1000 Genomes databases. In brief, 10–15 ng of DNA (1 μL), 5 μL of Master Mix for genotyping (TaqMan Genotyping Master Mix^®^—2×—Thermofisher Scientific, Waltham, MA, USA), 0.5 μL of specific primers and probes (TaqMan Genotyping Assay Mix^®^—20×—Thermofisher Scientific, Waltham, MA, USA), and 3.5 μL of DNAse- and RNAse-free water were added to each well of a qPCR plate, totaling a final volume of 10 μL for each sample. After qPCR amplification (first denaturation at 94 °C/5 min, 30 cycles of denaturation at 94 °C/10 s, annealing at 60 °C/30 s), the genotype was determined using StepOnePlus™ v2.3 software and confirmed using TaqMan^®^ Genotyper software (2020). The *BDNF* polymorphism was described using *C*–*T* allelic distribution according to the GRCh37.p13chr11 and GRCh38.p12chr11 versions found in the 1000 Genomes database. Details of the investigated SNPs are provided in the Supplementary Materials.

### 2.3. Statistical Analysis

Inferential statistics were performed using non-parametric tests considering the categorical nature of the values obtained from the Negative Addiction Scale questionnaire. The Chi-squared test was used to analyze the association between different distributions, with adjusted residuals considered to score the observed differences. The Mann–Whitney test was used to compare the scalar scores of different polymorphisms within the same gene. The scale values for each polymorphism are expressed as the median and interquartile range for the central tendency and dispersion tendency measures, respectively. A significance level of 5% (*p* < 0.05) was accepted for all inferential analyses. All statistical tests were performed using Statistical Package for the Social Sciences (SPSS) v20.0.

Hardy–Weinberg equilibrium was tested using the Genetics package for R software (4.0.1), which was checked by Arlequin software (3.5.2). For the genetic association analysis, we used the codominant model to understand all effects of every genotype globally. For central and dispersion values according to the exercise addiction score, we isolated the more ancestral allele to better understand its evolution over time.

The sample size was calculated based on a power of 80% and alpha of 0.05, considering Bonferroni correction and Cohen’s effect size and using the Chi-squared method of independence (χ^2^) of Cohen’s W for one SNP, like DRD2, and the exercise addiction phenotype.

## 3. Results

Of the 469 study participants, 240 reported practicing physical exercises regularly. Of these 240 physically active individuals, 98 (40.8%) reported symptoms suggestive of exercise addiction, represented by scores equal to or greater than five points on the Negative Addiction Scale. With the slight exception of BDNF (*p* = 0.044), all SNPs achieved Hardy–Weinberg equilibrium.

Table 1 shows the characteristics of the study sample. Ages and SNPs genotypes were homogeneous between the sexes. However, for all exercise-related variables, the practice, frequency, and volume of activities were significantly higher among the men.

In an initial analysis of the associations between symptoms suggestive of addiction and SNPs, which considered three possible allelic distributions (i.e., two homozygotic and one heterozygotic), only the *GG* genotype of the *DRD2* polymorphism showed a significant association with asymptomatic individuals. In contrast, the *AA* genotype of the same polymorphism was associated with symptomatic participants (Table 2, *p* = 0.027).

In the second analysis, the allelic distribution was divided into two groups, given the possibility that the presence of the *BDNF T* allele alone could increase the asymptomatic condition for exercise addiction. This grouping was based on the most ancestral allele C and the possibility of comparison of fewer groups. In this study, there was indeed a trend of an association of these two alleles with the asymptomatic condition.

The analysis of gene polymorphisms in the entire sample group according to the addiction scores revealed a significant difference between the *CC* and *TT*/*CT* (*p* < 0.05) genotypes of the *BDNF* gene concerning physical exercise addiction. In contrast, the *AA* and *GG*/*AG* genotypes of the *DRD2* gene showed no significant difference in this regard (*p* > 0.05) (Table 3).

The inferential analysis based on only the individuals who practiced physical exercise showed no significant difference regarding the addiction scores for either the *BDNF* or *DRD2* polymorphisms (*p* > 0.05) (Table 4). There were also no differences between the participant groups with regard to the *ACTN3*, *PPARA*, *HFE*, *DRD1*, *PPARGC1A*, and *AMPD1* SNPs studied.

## 4. Discussion

Physical exercise addiction is a compulsive behavior that can lead to withdrawal symptoms and other adverse effects, with biological and social factors being potential causes of its development [5,11]. Despite suggestions of a possible genetic involvement, as well, there are as yet no published genetic association studies indicating candidate genes for exercise addiction in humans. Thus, this study is the first to propose an approach for such an investigation, with the objective of genotyping the main *DRD1*, *DRD2*, *BDNF*, *PPARA*, *AMPD1*, *HFE*, *PPARGC1A*, and *ACTN3* gene polymorphisms previously associated with either physical activity performance or level and verifying whether they are associated with exercise addiction.

In total, 469 participants were enrolled for this genetic association study, and of these individuals, 28.2% (n = 67) showed indications for exercise addiction, corroborating the results reported by Nogueira et al. [5]. There was also a significant association of the *DRD2* rs1800497 polymorphism with exercise addiction individually, with the *A* allele being associated with symptomatic exercise addiction and the *GG* genotype being associated with asymptomatic outcomes, but without a multiple comparison support.

Physiologically, the *A* allele of this polymorphism appears to reduce the number of D2 receptors, thereby increasing the level and responsiveness of dopamine as a result of the lower inhibitory feedback of these receptors [7,17,18,19]. In this regard, the findings of this study are consistent with the physiological function of the *A* allele, given that high exercise levels have been associated with high dopamine levels [8,20,21].

Changes in dopamine levels have been suggested as an influencing factor in exercise addiction and in other compulsive behaviors [14]. The rs1800497 polymorphism of *DRD2/ANKK1* has been associated with an increased risk of the compulsive use of different drugs and psychoactive compounds [14,15]. The involvement of dopamine in these types of behavior may be due to its participation in the reward and pleasure system, where it affects mood and motivation [11].

In their analysis of the effects of exercise on dopamine receptors in rodents for 6 weeks, Robison et al. [22] reported decreased D1 receptors and increased D2 receptors in some brain regions after exercise, without changes in the dopamine active transporter (DAT) levels. In a similar study, Fisher et al. [23] showed increased DAT and dopamine serum levels and decreased DRD2 expression after 30 days in response to increased exercise intensity. Thus, a high intensity of physical exercise may influence dopaminergic responses, activating the reward and pleasure system, which partly explains physical exercise addiction.

The findings by Robison et al. [22] and Fisher et al. [23] of the association of decreased DRD2 synthesis with an increased risk of drug-seeking and other compulsive behaviors supports our finding of the association of the *A* allele (a variant of the *DRD2* polymorphism) with exercise addiction, considering that this allele acts to decrease DRD2 expression and, consequently, increase dopamine levels. In contrast, unlike the physiological effects of the polymorphic allele, the *G* allele appears to increase the synthesis of DRD2, thereby decreasing dopamine levels, suggesting a protective effect in this population when considering only the physiological impacts of the allele. Interestingly, in this study, we found an association of the *GG* genotype with participants who were asymptomatic for exercise addiction.

The *CC* genotype of the *BDNF* polymorphism was also associated with physical exercise addiction, according to the analyses based on means or medians individually, but not after multiple comparisons. This is compatible with the physiological functions of the gene, given that the *T* allele of this variant appears to decrease *BDNF* levels and change dopaminergic transmissions and is a risk factor for dementia [24]. Bryan et al. [25] found a significant association of the *TT* genotype with higher levels of exercise practice compared with the *CC* genotype. In contrast, this study found a significant association of the *CC* genotype with exercise addiction.

It is important to emphasize that the other six polymorphisms investigated in this study showed no significant associations with exercise addiction. However, they had been previously described as being associated with either physical exercise performance [10] or physical exercise levels [6,7,26]. Thus, those six genes were expected to be associated with exercise addiction, considering that they are related to energy expenditure, mitochondrial biogenesis, muscle structure, muscle contraction, and ATP restoration, among other essential aspects of physical exercise performance that can increase engagement and motivation to exercise. Moreover, given the dopaminergic effects of the *DRD1* gene polymorphism, it was expected to have a significant association with exercise addiction [6,27].

Finally, understanding the factors that may be involved in the development of compulsive exercise may help health professionals to establish clinical measures and better tools for the diagnosis of this behavioral disorder. Furthermore, it will allow further investigation of the extent to which physical exercise can prove to be helpful and therapeutic in the treatment of other mental health disorders, such as anxiety, depression, and drug withdrawal, as has been pointed out in the literature [3,4,5,28,29]. Although our sample achieved the significant size of almost 500 participants, the DDR2 SNP presented a different distribution among groups but not after genotype grouping. On the other hand, the BDNF SNP was associated with addiction only after grouping genotypes and fewer degrees of freedom among groups. Moreover, multiple comparisons and Bonferroni correction showed that the association just persisted when analyzing the variant individually, pointing to a slighter association or a substantial necessity to increase the sample size. Although we saw a slight variation in only one SNP equilibrium from seven total SNPs evaluated, this configuration represents a limitation in the results analysis. The associations found support the difficulty of studying variables related to behavioral phenotypes. The Chi-squared method of independence (χ^2^) of Cohen’s W suggested a sample size for the genotyping of 386 individuals, which is apparently adequate for our population. However, whole-genome association studies have been known to suggest *p*-values of less than 5 × 10^−8^. Thus, although apparently adequate, the results should be analyzed with caution

## 5. Conclusions

To the best of our knowledge, the present study is the first to present data on the association of genetic polymorphisms with exercise addiction in humans, with the SNP rs1800497 of the *DRD2* gene showing a significant correlation in this regard, but only for individual SNP analysis, independently of multiple comparisons. These data corroborate those of previous studies on the possible association of genetic variants with psychoactive substance addiction. The *CC* genotype of the *BDNF* rs6265 polymorphism was significantly associated with exercise addiction only, according to mean and median analyses in a genetic grouping model based on the most ancestral allele and also on an individual level. The literature suggests that decreased *BDNF* expression may be a risk factor for dementia and that dopaminergic transport changes may occur, with possible consequences arising in the reward and pleasure system. The associations found show the difficulty of studying variables related to behavioral phenotypes.

Genetic association data should be carefully interpreted and implemented owing to the multifactorial nature of a given behavior or disease. It is also important to recognize that such knowledge could reinforce the stigmatization that individuals with specific genetic characteristics experience, which can motivate them to perform some modalities to the detriment of their personal wellbeing. Finally, further investigations into these outcomes are recommended to shed light on the extent to which physical exercise can, in fact, improve the treatment of mental health disorders.

Because of the scarcity of the literature on exercise addiction, the information shared in this article contributes to our better understanding of this behavior. Having access to the genomic profile of exercisers can enable exercise prescription and a more careful look at overexercising, considering that there may be a portion of the population that is more susceptible to developing behavioral changes associated with exercise addiction. In this way, both the prescription of exercise and the monitoring of exercisers’ behavior are vital in ensuring healthy physical exercise practices.

## Figures and Tables

**Table 1:** Sample characterization.

			Sex				
	Total		Men		Women		*p*
Variables	n	%	n	%	n	%	Value
Age group							0.311
18 and 19 years old	93	19.8	33	35.5	60	64.5	
20 to 22 years old	139	29.6	51	36.7	88	63.3	
23 to 26 years old	115	24.5	51	44.3	64	55.7	
≥27 years old	122	26.0	55	45.1	67	54.9	
Practices physical exercise?							0.000
No	229	48.8	61	26.6	168	73.4	
Yes	240	51.2	129	53.8	111	46.3	
Frequency of practice							0.000
1 to 2 times/week	28	11.7	8	28.6	20	71.4	
3 to 5 times/week	137	57.1	62	45.3	75	54.7	
≥6 times/week	75	31.3	59	78.7	16	21.3	
Time exercising							0.000
<399 min/week	119	49.8	48	40.3	71	59.7	
≥400 min/week	120	50.2	80	66.7	40	33.3	
*ACTN3*							0.125
TT	89	19.0	42	47.2	47	52.8	
CT	218	46.5	78	35.8	140	64.2	
CC	162	34.5	70	43.2	92	56.8	
*AMPD1*							0.499
AA	3	0.6	1	33.3	2	66.7	
AG	62	13.2	21	33.9	41	66.1	
GG	404	86.1	168	41.6	236	58.4	
*BDNF*							0.130
TT	11	2.3	6	54.5	5	45.5	
CT	88	18.8	28	31.8	60	68.2	
CC	370	78.9	156	42.2	214	57.8	
*DRD1*							0.843
AA	31	6.6	14	45.2	17	54.8	
AG	184	39.2	75	40.8	109	59.2	
GG	254	54.2	101	39.8	153	60.2	
*DRD2*							0.647
AA	32	6.8	13	40.6	19	59.4	
AG	157	33.5	59	37.6	98	62.4	
GG	280	59.7	118	42.1	162	57.9	
*HFE*							0.294
GG	4	0.9	2	50.0	2	50.0	
CG	106	22.6	36	34.0	70	66.0	
CC	358	76.5	151	42.2	207	57.8	
*PPARA*							
CC	49	10.5	21	42.9	28	57.1	0.172
CG	194	41.5	87	44.8	107	55.2	
GG	225	48.1	81	36.0	144	64.0	
*PPARGC1A*							0.729
TT	37	7.9	13	36.1	24	64.9	
CT	185	39.7	74	40.0	111	60.0	
CC	244	52.4	102	41.8	142	58.2	

**Table 2:** Associations between genotypic polymorphism characteristics and exercise addiction.

	Symptoms Suggestive of Addiction				
	Asymptomatic (0 to 4 Points)		Symptomatic (5 to 14 Points)		*p*
Single-Nucleotide Polymorphisms	n	%	n	%	Value
*ACTN3*					0.900
TT (XX)	69	77.5	20	22.5	
CT (RX)	174	79.8	44	20.2	
CC (RR)	126	78.8	34	21.3	
*AMPD1*					0.819
AA	2	66.7	1	33.3	
AG	48	77.4	14	22.6	
GG	319	79.4	83	20.6	
*BDNF*					0.088
TT	9	81.8	2	18.2	
CT	77	87.5	11	12.5	
CC	283	76.9	85	23.1	
*DRD1*					0.523
AA	22	71.0	9	29.0	
AG	145	79.2	38	20.8	
GG	201	79.8	51	20.2	
*DRD2*					0.027
AA	19	61.3	12	38.7	
AG	130	82.8	27	17.2	
GG	220	78.9	59	21.1	
*HFE*					0.278
GG	4	100.0	0	0.0	
CG	88	83.0	18	17.0	
CC	276	77.5	80	22.5	
*PPARA*					0.093
CC	40	83.3	8	16.7	
CG	143	74.1	50	25.9	
GG	181	82.2	40	17.8	
*PPARGC1A*					0.136
TT	30	81.1	7	18.9	
CT	153	83.2	31	16.8	
CC	183	75.3	60	24.7	

**Table 3:** Central and dispersion values according to the exercise addiction score for each gene polymorphism in the entire sample group.

Gene	Polymorphisms		U	*p* Value
BDNF	CC	TT/CT		
	0.5 (0–4)	0 (0–3) *	15,506.000	0.014
				
DRD2	AA	GG/AG		
	2 (0–6)	0 (0–4)	5723.5	0.12

* Significant difference relative to the *CC* genotype of the *BDNF* gene.

**Table 4:** Central and dispersion values according to the exercise addiction score for each gene polymorphism in the exercise group.

Gene	Polymorphisms		U	*p* Value
BDNF	CC	TT/CT		
	4 (2.25–6)	4 (2–5)	3468.000	0.1
				
DRD2	AA	GG/AG		
	5 (2.75–8)	4 (2–6)	1455.5	0.06

## Data Availability

The data generated and analyzed during the study are available from the corresponding author on request.

## Supplementary Materials

The following supporting information can be downloaded at https://www.mdpi.com/article/10.3390/ijerph22091356/s1, Table S1: Summary of information on the SNPs investigated.
