# Supplementary material for: Association of DRD2 and BDNF Genetic Polymorphisms with Exercise Addiction
Source: Int J Environ Res Public Health. 2025 Aug 29;22(9):1356. doi: 10.3390/ijerph22091356 (PMC12469787; doi:10.3390/ijerph22091356)
Supplement: Supplementary file 1 [file ijerph-22-01356-s001.zip › ijerph-3774543-supplementary.pdf]

Table S1. Summary of information on the SNPs investigated.

Loci	Literature Overview	Tipo	dbSNP	Nucleotídeo	Mudança	Localização	Função
ACTN3	A gene encoding the protein alpha-actinin 3, which is essential in anchoring and stabilizing thin chains of actin to the Z-line. The variant results in the premature halt of protein synthesis and is commonly referred to as R577X.	NONSENSE	rs1815739	C ⇒ T	**C**GA ⇒ **T**GA *Arg* ⇒ X	chr11:66560624 (GRCh38.p12)	The premature *stop codon* generated due to the polymorphism results in the interruption of the synthesis of this protein. Thus, T/T individuals do not produce the alpha-actinin-3 protein.
AMPD1	A gene encoding the enzyme adenosine monophosphate deaminase 1, which is important for purine metabolism. The variant results in a premature stop codon and is commonly referred to as C34.	NONSENSE	rs17602729	C ⇒ T	**C**AA ⇒ **T**AA *Gln* ⇒ X	chr1:114693436 (GRCh38.p12)	It can generate a muscle deficiency of AMP deaminase. The T allele appears to decrease AMP deaminase activity.
*BDNF**	This gene encodes a member of the nerve growth factor family of proteins. The binding of this protein to its cognate receptor promotes neuronal survival in the adult brain. It may play a role in regulating the stress response and the biology of mood disorders. *Met* codes the A allele and *Val* the G allele.	MISSENSE	rs6265	C ⇒ T	**G**TG ⇒ **A**TG *Val* ⇒ *Met*	chr11:27658369 (GRCh38.p12)	Substitution of the amino acid Valine with Methionine results in lower neuronal expression of *BDNF*, lower hippocampal volume, memory, and impaired hippocampal activation.
DRD1	This gene encodes the D1 subtype of the dopamine receptor, the most abundant receptor in the central nervous system. This G-protein-coupled receptor stimulates the activity of adenylyl cyclase and cyclic AMP-dependent protein kinase. These receptors regulate neuronal growth and development and aid in some behavioral responses.	5' UTR	rs265981	A ⇒ G	N/A	chr5:175443899 (GRCh38.p12)	The A allele decreases the expression of the *DRD1* gene, resulting in lower dopamine levels.
DRD2	This gene encodes the D2 subtype of the dopamine receptor. This G-protein-coupled receptor inhibits the activity of adenylyl cyclase. In addition, it is known as TaqI *DRD2*.	MISSENSE	rs1800497	G ⇒ A	**G**AG ⇒ **A**AG *Glu* ⇒ *Lys*	chr11:113400106 (GRCh38.p12)	The G allele increases the expression levels of the *DRD2 gene*, which results in decreased dopamine levels. The A allele reduces the number of D2 receptors,

							which generates an increase in dopamine levels.
HFE	This gene encodes a protein membrane-associated with beta2-microglobulin, regulating iron uptake by controlling the interaction of transferrin receptors with transferrin (transporter).	MISSENSE	rs1799945	C ⇒ G<br>C ⇒ T	**C**AT ⇒ **G**AT<br>**C**AT ⇒ **T**AT	chr6:26090951 (GRCh38.p12)	Polymorphisms in this gene alter the body's absorption of iron. Individuals homozygous for the Cys282Tyr polymorphism may manifest hemochromatosis.
PPARA	Peroxisome proliferators induce an increase in the size and number of peroxisomes (cell organelles that participate in respiration and lipid metabolism), which can be regulated by PPAR receptors, affecting cell proliferation, cell differentiation, and immune and inflammatory responses.	INTRON VARIANT	rs4253778	G ⇒ C	N/A	chr22:46234737 (GRCh38.p12)	The C allele affects PPARα function and downregulates the activity of mitochondrial fatty acid oxidation enzymes, reducing their activity and impairing cellular lipid homeostasis.
PPARGC1A	It is the gene encoding PGC-1, a transcriptional coactivator involved in energy metabolism and mitochondrial biogenesis. The variant studied is commonly referred to as Gly482Ser, with the A allele encoded by 482Ser and the G allele by Gly482.	MISSENSE	rs8192678	G ⇒ A	**G**GT ⇒ **A**GT<br>*Gly* ⇒ *Ser*	chr4:23814039 (GRCh38.p12)	Genetic variations in this gene can cause a reduction in its activity with the expression of the Gly482Ser allele, which is associated with obesity.

*BDNF C-T polymorphism according to versions GRCh37.p13chr11 and GRCh38.p12chr11, but considered G-A according to reference sequence (BDNFRefSeqGene).
